# Utility of Diffusion Tensor Imaging in Assessing Corticospinal Tracts for the Management of Brain Tumors: A Cross-Sectional Observational Study

**DOI:** 10.7759/cureus.47811

**Published:** 2023-10-27

**Authors:** Deepmala K Karmakar, Padma V Badhe, Pauras Mhatre, Shashwat Shrivastava, Moinuddin Sultan, Gautham Shankar, Khushboo Tekriwal, Swapnil Moharkar

**Affiliations:** 1 Radiology, Seth Gordhandas Sunderdas Medical College and King Edward Memorial Hospital, Mumbai, IND; 2 Radiology, Vedantaa Institute of Medical Sciences, Dahanu, IND

**Keywords:** brain spine tumors, magnetic resonance (mr), neurologic prognosis, diffusion tensor imaging, white matter lesions

## Abstract

Introduction

Intra-axial brain tumors are a significant health problem and present several diagnostic and treatment challenges. Conventional magnetic resonance imaging (MRI) has posed several limitations, such as the inability to delineate the detailed anatomy of fibers in structures like the brainstem and the inability to accurately judge the extent of tumor infiltration. Diffusion tensor imaging (DTI), based on the concepts of isotropic and anisotropic diffusion, is capable of visualizing and segmenting white fiber bundles in high detail and providing crucial information about tumor boundaries, extent, neighboring tracts, and more. This information can be very useful in initial non-invasive diagnosis, preoperative tumor grading, biopsy planning, surgical planning, and prognosis.

Methods and materials

This is a cross-sectional observational study in a tertiary care setup, conducted over a one-year period. The study was performed in Seth Gordhandas Sunderdas Medical College (Seth G.S. Medical College) and King Edward VII Memorial Hospital (K.E.M. Hospital), a tertiary care hospital located in Mumbai, India. Fiber tractography was performed and was used to visualize the corticospinal tracts passing through the length of the brainstem. Changes in the degree of infiltration, destruction, and displacement of the corticospinal tracts were observed carefully. Adult patients who were diagnosed with brain tumors, willing to participate in the study, and capable of providing written informed consent prior to study registration were included. The DTI findings along with information from other investigations were used to decide the best course of management for each case.

Results

The study included 30 participants with a mean age of 46.0 ± 17.1 years, 63.3% and 37.7% being male and female, respectively. According to the lesion's location, the pons was found to be the most often affected area in 23.33% of cases, followed by the temporo-parietal region (13.3%) and the frontal region (13.3%). These lesions had heterogenous enhancement in 63.3% of the instances and homogeneous enhancement in 36.7% of the cases, according to a contrast study. According to their consistency, the lesions were further divided into two categories: solid lesions, which were present in 66.7% of instances, and cystic lesions, which were present in 90% of cases. Results from the diffusion tensor technique revealed that infiltration accounted for 40.0% of cases, displacement for 76.7%, and loss of white fiber tracts for 20.0%. DTI findings were significantly associated with the type of planned management and with the presence of post-management neurological deficit.

Conclusion

DTI played a complementary role in the assessment of tumors and can be used to improve surgical planning and therapeutic decision making. Preservation of corticospinal tracts is vital to prevent motor impairment. Availability of qualitative data with the depiction of corticospinal tracts in a three-dimensional projection and their relation with the brain tumors by DTI greatly helps in preoperative decision making and surgical approach.

## Introduction

The incidence of brain tumors in India ranges between five and 10 cases per 100,000 members of the population with an increasing trend and accounts for 2% of malignancies [1]. Central nervous system neoplasms are a histologically diverse group of disorders that occur at many sites in the brain or its linings [2]. Intra-axial brain tumors are a significant health problem and present several diagnostic and treatment challenges. These lesions include glioblastoma multiforme (GBM), astrocytoma, oligodendroglioma, and ganglioglioma [2,3]. Intra-axial brain tumors arise within the brain parenchyma as opposed to extra axial tumors that describe lesions outside the brain and intra-ventricular tumors that denote lesions within the ventricular system [3]. 

Conventional magnetic resonance imaging (MRI) has been extensively used for aiding the diagnosis of brain tumors, but there are significant limitations with the information that it provides [4,5]. Some of the limitations of conventional MRI are that it cannot delineate the detailed anatomy of fibers in structures like the brainstem, and it has similar display signal intensity for both high-grade gliomas (HGGs) and solitary metastasis and thus is not able to accurately characterize the extent of tumor infiltration [4]. Owing to this, we largely must rely on histopathological studies to come to an accurate diagnosis [2,6,7-15]. The most recent developments in brain tumor imaging provide novel anatomical and pathophysiological information that offers fresh perspectives on brain tumors and helps with therapy decisions and prognostic information [7-15]. Currently, this knowledge is applied in the clinical practice for prognostication; non-invasive, preoperative tumor grading; biopsy planning; surgery; and radiation portal planning [7-15].

The MRI brain protocol for tumors includes a diffusion-weighted MR scan as a key element [16]. Diffusion-weighted MR imaging is a non-invasive MRI-based neuroimaging technique that helps to estimate the location, orientation, and anisotropy of the brain’s white matter (WM) tracts [7-9]. Diffusion-weighted MR imaging is the simplest form of diffusion imaging. It is a pulse sequence sensitized to the random motion of water molecules (which is termed as Brownian motion) [7-9]. One can discriminate between protons diffusing quickly (known as unrestricted diffusion) and slowly (known as limited diffusion) using diffusion weighting [7-9]. On diffusion pictures and the corresponding apparent diffusion coefficient (ADC) maps, lesions with limited diffusion show hyperintensity [7-9].

Diffusion tensor imaging (DTI) is a more advanced technique of diffusion imaging [7-9]. The sole non-invasive, in-vivo approach for mapping the WM tract trajectories in the human brain is diffusion tensor MR imaging [7-15]. The ideas of isotropic and anisotropic diffusion serve as the foundation for DTI [7-15]. Isotropic diffusion occurs when water molecules diffuse equally in each of the three directions [7-15]. This is typically observed in ventricles and the gray matter. Meanwhile, if water diffusion is unequal in all three directions, then it is called as anisotropic diffusion and is observed in the WM [7-15]. In WM tracts, the myelin sheath surrounding the WM causes the water molecules to move more along the long axis of a fiber bundle and less perpendicularly [7-15]. WM tracts around tumors are characterized as displaced, disrupted, infiltrated, and edematous [15].

Diffusion-weighted imaging (DWI) and DTI are used to characterize mass lesions in brain tumors that are frequently unspecific on conventional MRI and to help in differential diagnosis [17]. On fluid-attenuated inversion recovery (FLAIR) and T2-weighted imaging, low-grade (LGGs) and high-grade (HGGs), metastases, and lymphomas exhibit varying high signal, although they are hypo- to isointense on T1-weighted images [18]. Contrast-enhanced T1-weighted imaging may reveal some degree of enhancement in the majority of lesions [18]. It might be challenging to distinguish between non-tumoral lesions, such as acute and subacute infarcts, tumefactive demyelinating lesions, abscesses, and hematomas [18]. Accurate diagnosis may be achieved by combining more sophisticated neuroimaging methods, such as DWI and DTI, with MR spectroscopy, perfusion, and permeability imaging [18].

DTI aims to aid the management and assessment of brain tumors with its ability to visualize and segment white fiber bundles opening new perspectives for neurosurgeons [7-15]. Determination of tumor boundaries and assessment of tumor extension also provides useful information for tumor grading, follow-up, and monitoring disease progression [2]. In the present study, we demonstrated how information from DTI can be used to delineate the relationship between brain tumors and adjacent WM tracts to aid preoperative planning and improve surgical outcomes. Moreover, we evaluated the role of DTI in the assessment of different patterns of WM involvement (infiltration, displacement, and destruction) in patients with brain tumor.

## Materials and methods

The study was conducted across a period of one year from November 2018 to November 2019 by the Department of Radiology in Seth Gordhandas Sunderdas Medical College (Seth G.S. Medical College) and King Edward VII Memorial Hospital (K.E.M. Hospital), a tertiary care hospital located in Mumbai, India. This was a non-interventional and cross-sectional study. Thirty cases were studied, and both male and female participants were included. The study included adult patients who were diagnosed with brain tumors, were willing to participate in the study, and could provide written informed consent prior to study registration. The patients not willing to participate in the study or unable to tolerate MRI due to various reasons, such as claustrophobia or pacemaker or aneurysm clip, were excluded. Pregnant or nursing females, children, patients with retarded mental condition, and patients with kidney dysfunction (serum creatinine >2 mg/dL) were also excluded from the study.

Written informed consent was obtained from the patients after explaining to them about the possible risks and benefits of the examination. Essential clinical history was obtained mainly regarding previous surgery or interventional procedure. All the study related data were collected at the time of undergoing MRI scan. The patients did not have to visit the tertiary care center solely for the purpose of this study. DTI is a specialized sequence built into the MRI machine. There are a variety of protocols in the MRI machine, one of which is the brain tumor protocol [16]. It is performed in every patient with intracranial space occupying lesions. The reports of all imaging procedures were made available to the participants, and the evaluation of these was an integral part of the MRI report.

Imaging technique

Multi-echo multi-planar MR brain was performed on Siemens MAGNETOM Aera 1.5T scanner (Siemens Healthineers, Germany). Standard MRI brain includes (i) acquiring the localizer; (ii) T2 turbo spin echo (TSE) 3 mm axial images; (iii) T2 FLAIR coronal 3 mm images; (iv) DWI; (v) pre-contrast T1 TSE axial images; and (vi) post-contrast T1 TSE (FLAIR) coronal, sagittal, and axial 3 mm images.

MR perfusion is a part of our routine tumor protocol but was excluded in this study. The routine sequences were followed by additional sequences for DTI of four minutes and 24 seconds, which include (i) T1 magnetization-prepared rapid gradient echo (MPRAGE) and (ii) DTI echo planar imaging (EPI) sequence (field of view (FOV): 230 mm, slice thickness: 5 mm, repetition time/TR: 3500 ms, time to echo/TE: 83 ms, slices: 22).

The entire scan took around 30 minutes, and all the images obtained were sent to the workstation electronically for analysis. Gadopentetate dimeglumine (Magnevist) contrast was used with a dosage of 0.1 mmol/kg body weight. Post-processing of the DTI was performed using in-house Tractography Syngo software by Siemens. Three-dimensional (3D) anatomy series and EPI DTI tensor datasets were loaded into the Neuro 3D Task Card. The clip plane was activated, and seed points drawn over the area of desired tracts and tractography were generated. Using visual inspection and a WM atlas as a reference, the major WM pathways running through the supratentorial and infratentorial compartments of the brain were identified on each fractional anisotropy (FA) color map. To identify regions of interest (ROIs), the left and right corticospinal tracts were divided into ROIs. If the tract could not be reliably identified with the brain, a ROI was placed in the approximate area of the tract. 

Fiber tractography was performed and was used to visualize the corticospinal tracts passing through the length of the brainstem. Superiorly, the corticospinal tract was visualized as far as practicable above the level of the corpus callosum. Changes in the degree of infiltration, destruction, and displacement of the corticospinal tracts were observed carefully. The fibers were color-coded according to their direction, red for fibers crossing left to right and vice versa, green for fibers traversing in the antero-posterior direction, and blue for fibers in the superio-inferior axis. Tracts with oblique angles were represented by a mixture of colors. The DTI findings along with information from other tests were used to decide management options for individual patients along with information from other tests, such that post-operative neurological deficit was minimized. Surgery was avoided if the patient had already developed neurological deficit. The presence of post-management neurological deficit four weeks after management (both surgical and conservative options), as assessed by clinical neurologists and neurosurgeons in the institute, was recorded in the study. 

Statistical analysis

Data were collected and compiled using the EPI Info software (version 7.2; by Centers for Disease Control and Prevention). All the qualitative data were expressed in terms of numbers and percentages. The quantitative data were either expressed in terms of the mean and standard deviations or categorized into groups and expressed in terms of percentages. The difference between the two proportions was tested using the Freeman-Halton extension of Fisher’s exact test. Analysis was two-tailed, and the significance level was set at 0.05.

## Results

The study comprised of 30 subjects with a mean age of 46.0 ± 17.1 years, from which 63.3% subjects were male and 37.7% were female. Based on the location of the lesion, the most common area involved was observed to be pons in 23.33% of the cases, followed by temporo-parietal in 13.3% and frontal in 13.3% cases. A contrast study of these lesions showed heterogenous enhancement in 63.3% of the cases and homogenous enhancement in 36.7% of the cases. The lesions were also classified as solid and cystic based on their consistency, and 66.7% of the cases showed solid lesions and 90.0% showed cystic lesions. Diffusion tensor findings showed that 40.0% of the cases were of infiltration and 76.7% of displacement, and 20.0% had destruction of white fiber tracts. Table 1 and Table 2 give a statistical summary of management methods used for different types of lesions and presence of post-management neurological deficit in the three types of white fiber tract lesions. Statistical analysis shows that there is a significant association of infiltration of white fiber tracts with the type of planned management and with the presence of post-management neurological deficit and that of destruction of white fiber tracts with the type of planned management.

**Table 1:** Association of DTI findings of the types of white matter involvement with the type of management used. *p<0.05 is significant. **Conservative management involved less aggressive surgical techniques, such as debulking without complete tumor removal.

DTI findings	Type of management		
	Surgical (n=18)	Conservative** (n=12)	p-value
Infiltration	4 (22.2%)	9 (75.0%)	0.008*
Displacement	16 (88.9%)	7 (58.3%)	0.0837
Destruction	0 (0.0%)	5 (41.7%)	0.0056*

**Table 2:** Association of DTI findings of the types of white matter involvement with post-management neurological deficit. *p<0.05 is significant.

DTI findings	Post-management neurological deficit		p-value
	Present (n=18)	Absent (n=12)	
Infiltration	12 (66.7%)	1 (8.3%)	0.0024*
Displacement	12 (66.7%)	11 (91.7%)	0.1931
Destruction	5 (27.8%)	0 (0.0%)	0.0657

## Discussion

Among the primary brain tumors, adults most frequently develop diffusely infiltrating gliomas, which range in severity from low grade (WHO grade II) to high grade (WHO grades III and IV). This tumor grade is based on histological findings. For the purposes of therapeutic planning, prognostication, and therapeutic response, it is crucial to differentiate between HGGs and LGGs [19]. Since tumoral enhancement is primarily caused by disturbance of the blood-brain barrier and not tumor angiogenesis, the appearance of contrast enhancement on CT and conventional MRI is not a reliable finding [5]. On a typical MRI, imaging results in brain tumors are usually unspecific. On FLAIR and T2-weighted imaging, both LGG and HGG gliomas exhibit varying high signal, whereas they are hypo- to isointense on T1-weighted images, and all lesions may exhibit some degree of enhancement on contrast-enhanced T1-weighted images [18].

It can often be challenging to distinguish between tumors and non-tumoral lesions, such hematomas, abscesses, and tumefactive demyelinating lesions [18]. In a study by Kono et al., 50.0% of patients with diffuse astrocytoma grade II had aberrant contrast enhancement [20]. Meanwhile, a significant number of supratentorial gliomas revealed that one-third of the non-enhancing tumors were cancerous [5,21]. The presence of peritumoral edema, mass effect, tumor heterogeneity, central necrosis, and intra-tumoral bleeding are additional observations in HGGs on conventional MRI [22]. DWI and DTI serve to characterize mass lesions to aid in differential diagnosis. In situations when conventional MRI falls short, DWI and DTI, together with MR spectroscopy, perfusion, and permeability MR imaging, may be useful in making an accurate diagnosis. With its capacity to see and segment white fiber bundles, DTI therefore seeks to completely transform the management and evaluation of brain tumors.

McKinney (2004) had studied the incidence of brain neoplasms in all age groups and found that primary brain neoplasms occur most commonly in the seventh decade of life [23]. This matches with the observation in our study where brain neoplasms were found mostly in patients above 50 years of age (53.3%), followed by age groups 40-50 years and 20-30 years comprising 13.3% of total cases each. Out of 30 patients, the incidence of brain neoplasms was more in males (n = 19) than females (n = 11). This agreed with the findings about sex differences in brain tumors done by Sun et al., where it was found that the incidence was more in males than females [24]. 

A study by Phuong et al. showed that the pons was the most common location of brainstem gliomas [25]. This was reflected in our study, where 23.3% (n = 7) neoplasms were located in the pons, making it the most common location of infratentorial brain tumors. The incidence of gliomas in the supratentorial-temporal region was also 23.3% (n = 7). The second most common locations were the temporo-parietal region 13.3% (n = 4) and frontal lobe 13.3% (n = 4). Larjavaara et al. found that the gliomas were most commonly located in the frontal lobe, followed by temporal and parietal lobes [26]. 

In our study, 63.3% (n = 19) cases showed heterogenous enhancement and 36.7% (n = 11) showed homogenous enhancement. A study published in the American Journal of Neuroradiology (2006) showed that high-grade tumors mostly show heterogenous enhancement and homogenous enhancement is mostly depicted by benign and low-grade lesions [27]. 

Jellison et al. [15] described four major patterns in affected WM tracts, namely, displacement, destruction, edematous changes, and infiltration, categorized on the basis of anisotropy and fiber direction or orientation. However, in our study, none of the cases showed an edematous pattern of WM involvement. Displacement, destruction, and infiltration were the only patterns observed in our study. 

**Figure 1:**
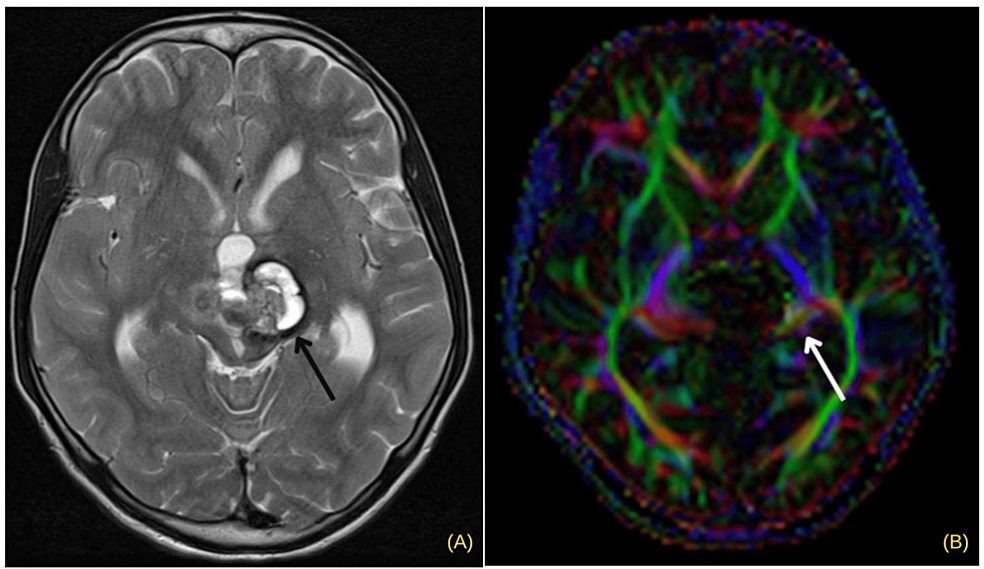
Imaging in a case of midbrain cavernoma with abnormal hues as seen on fractional anisotropy (FA) maps: (A) axial T2; (B) diffusion tensor imaging (DTI) T2 hyperintense lesion is seen in the left half of the midbrain, with hemosiderin rim (black arrow). Diffusion tensor imaging (DTI) fractional anisotropy (FA) map reveals displacement of corticospinal tracts with abnormal hues (white arrow).

Among the 23 cases of brain tumors showing displacement on DTI, only 16 were operated with favorable outcome. These cases showed benign characteristics on conventional MRI and displacement of corticospinal tracts. In a case shown in Figure 1, along with the displacement of the tracts, there were abnormal hues, and subsequently the patient had developed neurological deficit. Displacement of WM tracts is suggestive of a benign lesion; it confirms the presence of an intact peritumoral tract that can potentially be preserved during resection and thus can be of extreme help to neurosurgeon in preoperative planning [15].

**Figure 2:**
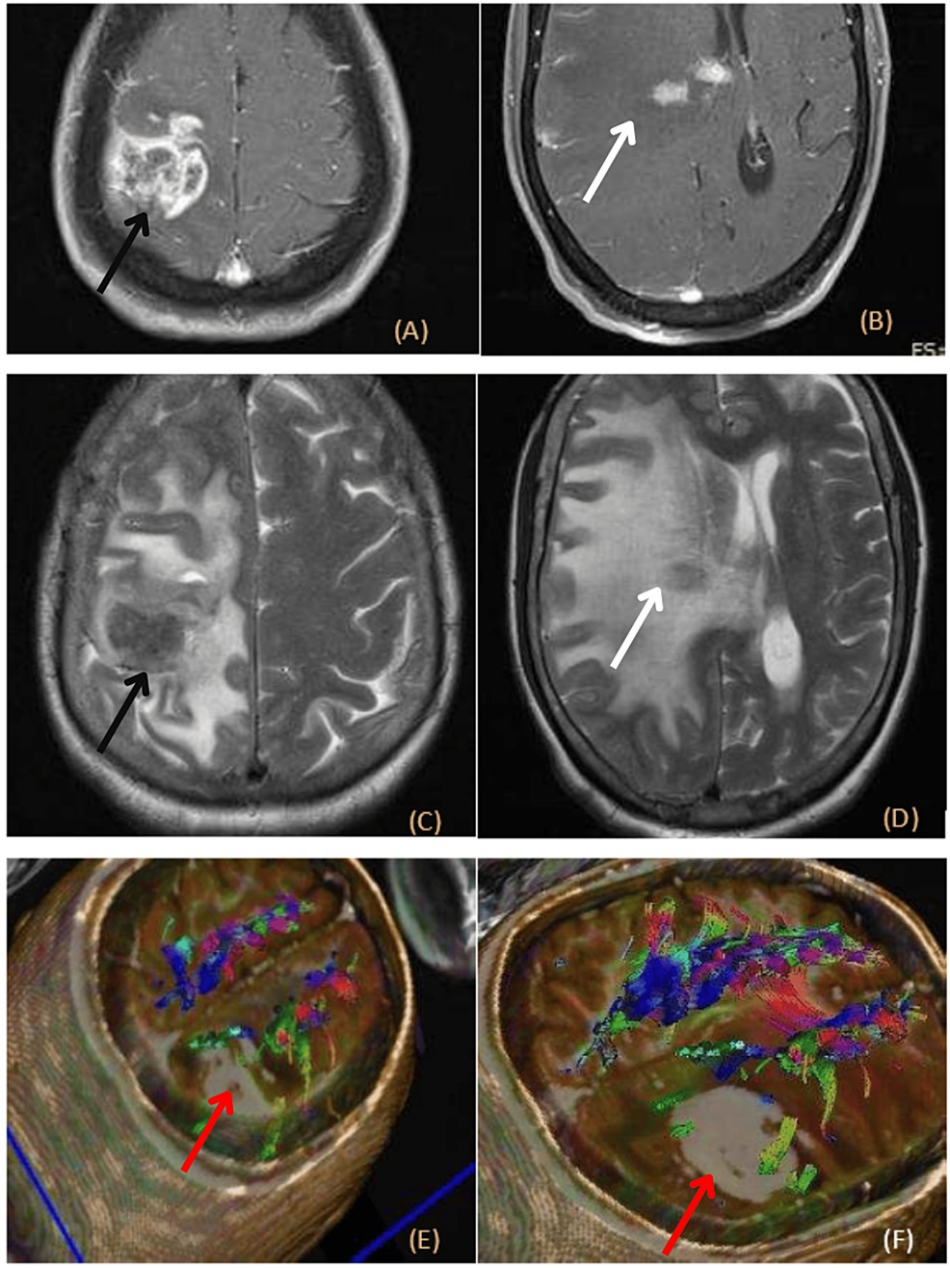
Imaging in a case of glioblastoma multiforme: (A, B) axial T1 post contrast; (C, D) axial T2; (E, F) diffusion tensor imaging (DTI) T2 heterogenous hyperintense lesion is seen in the right frontoparietal region with peripheral ring of enhancement (black arrows). Similar morphology lesion is also seen in the right internal capsule and body of corpus callosum (white arrows). DTI reveals destruction of the right corticospinal tracts (red arrows).

In three cases of GBM, conventional MR sequences showed heterogeneous hypo-intensity on T1W and heterogeneous hyperintensity on T2W imaging. Complete destruction of the corticospinal tracts was seen on DTI (Figure 2). Destruction of WM tracts is a definite characteristic of a high-grade neoplasm. The importance of destruction of the tracts on DTI lies in the fact that the neurosurgeon does not have to take special care in preserving the tract during resection of the brain tumor [15]. However, in our study, none of the patients showing destruction on DTI were subjected to surgery due to extreme preoperative morbidity and significant neurological deficit. Surgery would not have proved beneficial, so they were managed conservatively. In a case of high-grade glioma (GBM), there was extensive infiltration of the cortical spinal tracts along with majority of the tracts being displaced. The patient had developed neurological deficit and hence was managed conservatively. 

**Figure 3:**
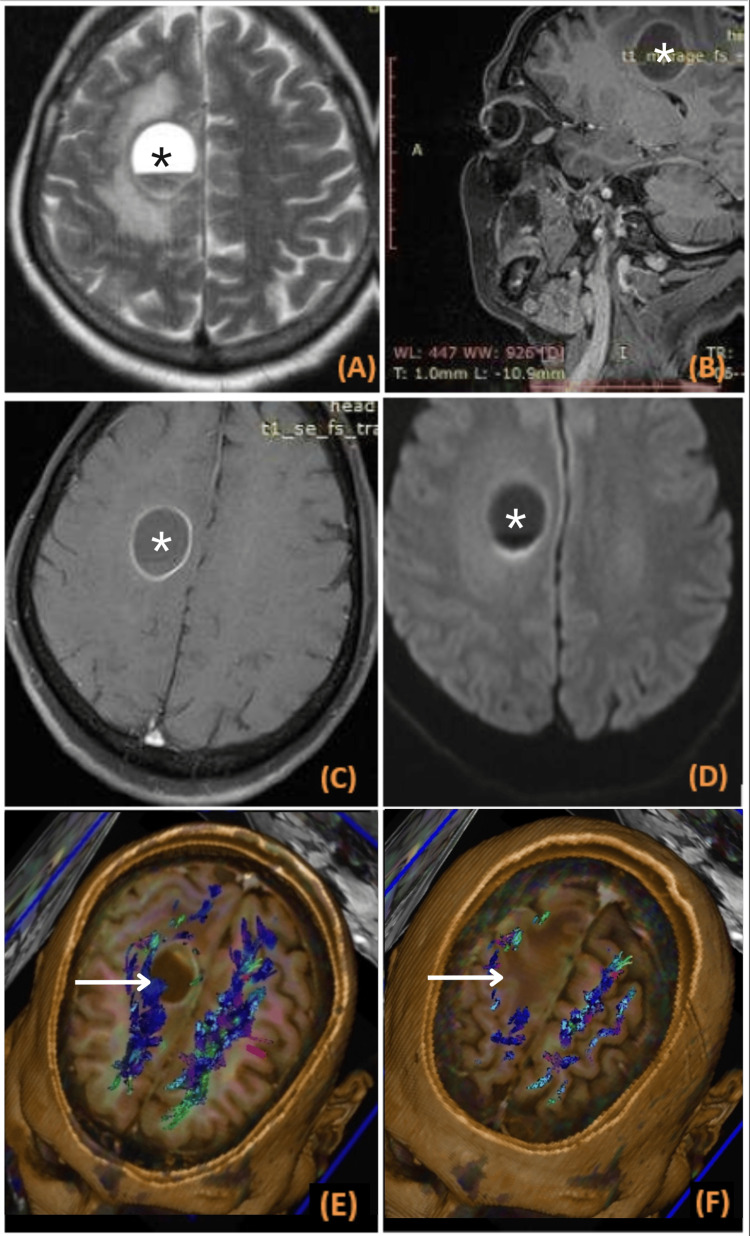
Imaging in a case of frontal glioma: (A) axial T2; (B) sagittal T1 post contrast; (C) axial T1 post contrast; (D) diffusion-weighted imaging; (E, F) diffusion tensor imaging A well-defined round predominantly T2 hyperintense lesion is seen (marked with asterisk *) with peripheral rim enhancement on post-contrast images and peripheral diffusion restriction on the DWI image. DTI reveals displacement with partial infiltration of the corticospinal tracts (white arrows).

In a case of frontal glioma and a case of diffusely infiltrating glioma, the DTI revealed partial infiltration of the corticospinal tracts along with displacement (Figure 3). Both patients were operated and had mild postoperative neurological deficit. In a case of high-grade pontine glioma, DTI revealed infiltration and posterior displacement of the left corticospinal tract. Hemorrhage, enhancement, and diffusion restriction were features in favor of a high-grade tumor. Infiltration of the corticospinal tract and its proximity to tumor margin on DTI were major factors in deferring surgery for this patient. The patient had already developed neurological deficit, and surgery would not prove beneficial; thus, the patient was managed conservatively. 

Jellison et al. speculated that an infiltrating tumor disrupts the directional organization of fiber tracts to cause altered color patterns on the directional map [15]. Infiltration along with displacement of the WM tracts is also indicative of a high-grade neoplasm. For patients with partial infiltration of the WM tracts, surgery can be attempted as there is minimal post-operative neurological deficit. 

As seen in most cases, the relationship of corticospinal tract with the space occupying lesion was most important for predicting neurosurgical outcome. In preoperative planning, DTI helps surgeons to know the relationship between the tumor and the surrounding WM tracts. To minimize the postoperative neurological deficit, the surgeon needs to know the exact tumor boundaries. In the above cases, DTI fiber tracking helped in the depiction of the lesion with relation to WM tracts and was an important factor in contributing to surgical decision making. In cases where a surgical approach was adopted, the surgeon depended on the exact location of corticospinal tracts to ensure resection without causing any damage to the tracts. Successful surgical outcome in those cases was possible due to the availability of detailed anatomical information provided by tractography.

To our knowledge, this is one of the initial studies conducted in a tertiary care public hospital in India, thus providing novel insights for the population of the country. With a fairly good sample size relative to the incidence of the disease, this study tried to highlight the importance and utility of DTI in delineating the anatomy of brain tumors and the related treatment implications. However, the study faces some limitations. Due to the cross-sectional study design, the study focused mainly on describing the DTI findings in patients with brain tumors. The superiority of the technique could only be proved by a comparative study, which provides further scope for research. Furthermore, long-term follow-up and prognosis could not be described. Considering the importance of surgical factors, such as histopathology, cancer driver mutations, and extent of resection, in the prognosis of patients with brain tumors, further research with longitudinal study designs is necessary.

## Conclusions

Brain tumors in adults are a significant health problem. Timely management of these and definitive delineation of WM tracts is crucial to avoid devastating neurological deficits. Preoperative mapping of corticospinal tracts is very important for predicting neurosurgical outcome since preservation of motor function is paramount. Depending on the extent and location of fiber disruption the motor impairment can be devastating. Availability of superior qualitative data with the depiction of corticospinal tracts in a 3D projection and the relation with tumor greatly helps in the management of brain tumors.

When the features observed in conventional MRI suggest tumors to be of high grade, DTI plays a complementary role in the assessment of the tumor and can be used to improve surgical planning and therapeutic decision making. DTI data must be interpreted as complementary to information from conventional MRI and other multiparametric techniques, such as MR perfusion and MR spectroscopy. A combination of these findings when correlated provides a much more detailed diagnosis and facilitates the choice of best treatment modality.
